# Pneumatosis Intestinalis in a Patient With Asthma: A Case Report

**DOI:** 10.7759/cureus.22116

**Published:** 2022-02-11

**Authors:** Tasniem Tasha, Priyata Dutta, Basher Atiquzzaman

**Affiliations:** 1 Internal Medicine, Rajshahi Medical College, Rajshahi, BGD; 2 Physiology, University of Michigan, Ann Arbor, USA; 3 Internal Medicine, University of Central Florida, Orlando, USA

**Keywords:** colonic wall, screening colonoscopy, gastrointestinal (gi), asthma, pneumatosis intestinalis

## Abstract

Pneumatosis intestinalis (PI) is a rare condition marked by gas-filled cysts in the submucosa and subserosa of the intestine. It can be idiopathic or linked to several illnesses, including gastrointestinal, pulmonary, collagen vascular disease, organ transplantation, and immunodeficiency. Herein we present a relatively rare case of PI in a 74-year-old man with a childhood history of asthma, which was found during routine colonoscopy.

## Introduction

Pneumatosis intestinalis (PI) is characterized by gas-filled cysts in the intestinal submucosa and subserosa. It is a rare disease, and the incidence rate is 0.03% in the general population [1]. PI was first described by DuVernoi in 1783 and later subclassified by Koss in 1952 [2]. It is classified into primary or idiopathic type (15%), and secondary type (85%) caused by various predisposing factors [3-4]. The secondary type consists of various etiologies like gastrointestinal, pulmonary, collagen vascular, organ transplantation, and immunodeficiency [2, 5]. PI can occur anywhere in the gastrointestinal tract distal to the stomach. In a retrospective review of 97 patients, the location of pneumatosis was as follows: colon 46%, small bowel 27%, stomach 5%, and 7% in both small and large intestines [6]. Mostly subserous cysts are present in the small bowels; on the contrary submucous localizations prevail in the colon wall [7]. Most cases of PI are asymptomatic and never come to medical attention. The most common manifestations in patients with small intestinal pneumatosis are vomiting, abdominal pain, weight loss, and diarrhea. On the other hand, diarrhea, hematochezia, abdominal pain, abdominal distension, and constipation are the most common symptoms of large intestinal pneumatosis [7-8]. PI associated with asthma is extremely rare. In this report, we present a case of PI associated with asthma in an otherwise healthy adult, which was detected during the routine colonoscopy.

## Case presentation

**Figure 1:**
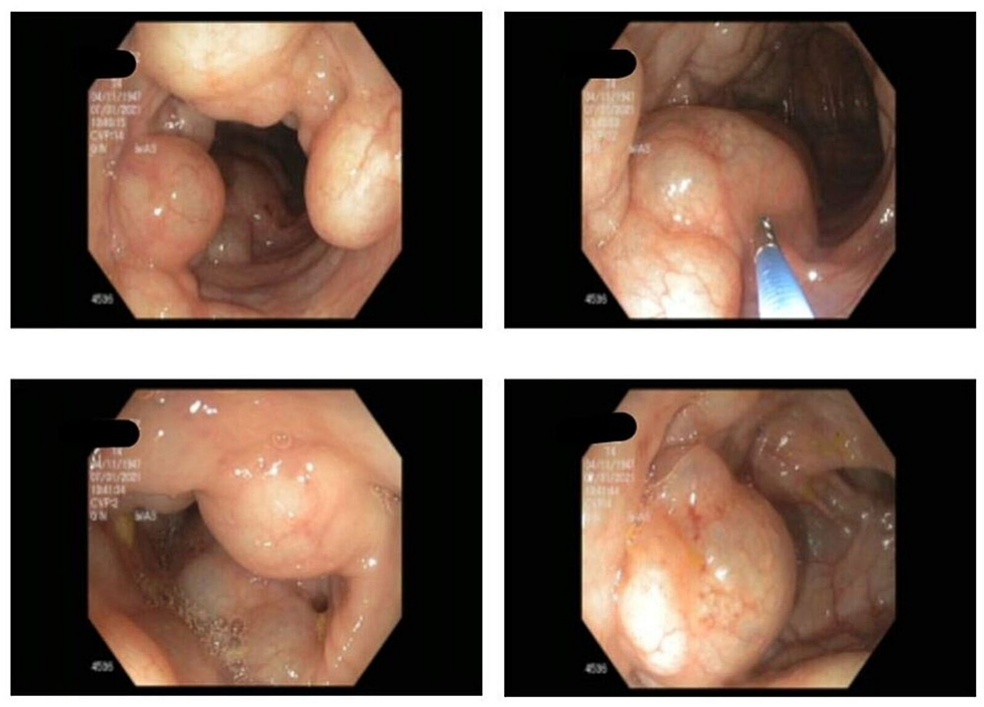
Colonoscopic images of PI at the mid and distal descending colon. PI, pneumatosis intestinalis

A 74-year-old Hispanic man presented with a medical history of well-controlled asthma since childhood to the gastroenterology outpatient clinic in Florida with the complaint of abdominal bloating for three months without any significant clinical history of abdominal distention, abdominal pain, bloody stool, tenesmus, or weight loss. A physical examination revealed no abnormalities. He had a history of smoking one pack per day for 20 years and has a history of asthma from childhood which is well controlled by anti-asthmatic medications (fluticasone-formoterol inhaler). A screening colonoscopy revealed the existence of multiple submucosal nodular projections in the wall of descending colon, some of which were transparent (Figure 1). Therefore, the diagnosis of PI was made. After being punctured by the needles, the bubbles collapsed, and the mucosa was removed and sent for histopathological examination. The patient’s symptoms resolved within three months after conservative medical treatment (antibiotics, elemental diet). On six months of follow-up, his gastrointestinal symptoms had been resolved and he remained asymptotic.

## Discussion

Pneumatosis intestinalis is a rare disease and is still poorly understood. Although previously PI was associated with postoperative complications of abdominal surgery, the prevalence has been increasing due to greater use of colonoscopy. It typically presents in the fifth to eighth decades of life [6, 9].

The pathogenesis of PI is yet to be clearly understood, however, it is considered multifactorial. Some cases of PI are incidental findings associated with a benign etiology, whereas in others, it presents as a life-threatening condition.

The primary type which is not associated with other diseases accounts for 15% of cases, and the secondary type accounts for the remaining 85% of cases [3-4]. Some predisposing factors that are associated with PI are mentioned in Table 1 [2, 5, 8]. 

**Table 1:** Common factors associated with PI. PI, pneumatosis intestinalis; SARS-CoV-2, severe acute respiratory syndrome coronavirus 2; AIDS, acquired immune deficiency syndrome

Types	Factors
Pulmonary disorders	Asthma, chronic obstructive pulmonary disease, cystic fibrosis, positive pressure mechanical ventilation
Intra-abdominal complications	Intestinal ischemia, infarction, obstruction, necrotizing enterocolitis, toxic megacolon
Mucosal disruption	Peptic ulcer disease, Crohn’s disease, ulcerative colitis, abdominal surgery, gastrointestinal endoscopy, ionizing radiation
Infections	*Clostridium difficile*, tuberculosis, AIDS-associated enterocolitis (cryptosporidium, cytomegalovirus, mycobacterium avium), SARS-CoV-2
Immunological disturbance	AIDS, steroid, amyloidosis, scleroderma, solid organ transplantation, lymphoproliferative disorder

Gas dissects into the intestinal wall from either the luminal surface through breaches in the mucosa or through the serosal surface by tracking along mesenteric blood vessels, according to the mechanical hypothesis. Once, inside the bowel wall, the gas might advance along the mesentery to distant locations. The mechanical theory explains the association of PI with obstructive pulmonary diseases. Coughing in these patients may induce alveolar rupture, allowing air to travel through blood vessels into the mediastinum, diaphragm, and finally to the mesenteric root. Once air has obtained access to the mesenteric root, it may travel via the mesenteric blood vessels and eventually through the intestinal wall [9-10]. In our case, the patient has a long-standing history of chronic asthma without other predisposing factors. We assumed that the severe cough because of asthma caused an abrupt increase in the intraluminal pressure in the intestinal wall susceptible to mechanical injury, leading to breaking in the mucosa of the intestinal wall and PI developed [10].

Bacterial theory reports that PI is caused by gas-forming bacteria getting access to the submucosa via breaks in the mucosa. This theory can be explained by the fact that the gas-forming bacillus *Clostridium perfringens* is linked to PI, and that this can be resolved with antibiotic treatment. Luminal bacteria produce excessive amounts of hydrogen gas through the fermentation of carbohydrates and other foods, according to biochemical theory. Gas can flow right through the mucosa and be trapped in the submucosal tissue when the pressure of the gas within the intestinal lumen rises [11-12].

The most common symptoms of PI include diarrhea, stomach pain, abdominal distension, loss of appetite, weight loss, constipation, flatulence, and tenesmus; however many individuals may be asymptomatic [5]. In most cases, such as ours, physical examinations reveal no significant symptoms or aberrant results. Simple X-rays, abdominal sonography, CT, and colonoscopy are all common diagnostic techniques for PI.

Patients who are asymptomatic do not need to be treated. The underlying cause of PI should be treated regardless of the appearance of symptoms. Antibiotics and an elemental diet may be considered for patients with mild symptoms [12-15]. Inhalation of oxygen therapy, antibiotics, and an elemental diet is used in the case of moderate to severe symptoms. Hyperbaric oxygen therapy can be provided for three days to patients who have persistent symptoms [16-17]. Furthermore, surgery is reserved for patients who continue to have symptoms despite medical treatment or who develop intestinal obstruction or perforation due to PI [3].

## Conclusions

Pneumatosis intestinalis (also called intestinal pneumatosis, pneumatosis cystoid intestinalis, pneumatosis coli, or intramural bowel gas) is pneumatosis of an intestine, that is, gas cysts in the bowel wall. The pathogenesis of PI is poorly understood and is likely multifactorial. This patient was diagnosed with asthma from his early childhood that was well controlled with anti-asthmatic medications. He presented with abdominal bloating and was followed by routine colonoscopy for further evaluation and was diagnosed with PI. In our case, we tried to highlight the association of asthma with PI according to the mechanical theory. PI is a rare condition, however, if a patient with a chronic pulmonary disease such as asthma presented with abdominal symptoms like abdominal bloating as in our case should arise suspicion of PI.
